# Letter to the editor regarding: “Long-term prognostic value of thyroid hormone levels in chronic critical illness patients”

**DOI:** 10.1080/07853890.2025.2537351

**Published:** 2025-07-21

**Authors:** Areeba Eeman Ahmad, Ayesha Shaikh, Syeda Mareeha Fatima

**Affiliations:** Federal Medical and Dental College, Islamabad, Pakistan

We read with great interest the article ‘Long-term prognostic value of thyroid hormone levels in chronic critical illness patients’ by Li et al. [1]. We applaud the authors for developing a new predictive model to predict 90-day mortality in CCI patients that outperforms the traditional scoring systems like SOFA and APACHEII. The authors concluded that decreased serum FT3 and FT4 levels in the first 10 days of ICU admission are associated with early mortality. It is praiseworthy that the study opens avenues for future interventional research to enhance the management of CCI patients affected by NTIS.

However, certain shortcomings should be acknowledged to provide a balanced perspective. We, therefore, would like to call attention to two significant points that can greatly impact the findings and may have been overlooked.

First, the decision to begin all thyroid hormone level measurements on day 10 post-ICU admission has halted the observation of early hormone fluctuations and dynamics that play a key prognostic factor for CCI patients. FT3 levels have been shown to fluctuate significantly in the initial days of critical illness, with day 5 as the optimal time point for predictive efficacy. Moreover, studies have shown that thyroid hormone levels may fluctuate more frequently depending on the disease severity, making a weekly measurement interval a potential limitation. These findings suggest that earlier and more frequent monitoring of thyroid hormones could offer a better assessment of NTIS progression and its prognostic value [2].

Secondly, the study population predominantly included patients with comorbid conditions such as hypertension, diabetes, and congestive heart failure. This raises the concern of whether the observed low T3 and T4 levels are specifically associated with these underlying conditions, rather than generalized to all patients with chronic critical illness. Employing a stratified approach could have provided a clearer understanding of the thyroid hormone patterns across different subgroups within the chronic critical illness population. Notably, low T3 and T4 levels have been identified as strong predictors of mortality in patients with heart disease, which may confound the interpretation of their prognostic value in a broader critically ill population [3].

In conclusion, while the study by Li et al. provides valuable insight into the association between decreased thyroid hormone levels and mortality in CCI patients, future research should refine methodological approaches to ensure more specific and accurate findings. Earlier and more frequent hormone monitoring, along with stratification based on comorbidities, may offer a clearer understanding of the prognostic role of thyroid dysfunction in this complex patient population.

## Data Availability

Data sharing does not apply to this article as no data was created or analyzed in this study.
